# COVID-19–Associated Misinformation Across the South Asian Diaspora: Qualitative Study of WhatsApp Messages

**DOI:** 10.2196/38607

**Published:** 2023-01-05

**Authors:** Anjana E Sharma, Kiran Khosla, Kameswari Potharaju, Arnab Mukherjea, Urmimala Sarkar

**Affiliations:** 1 Department of Family and Community Medicine University of California San Francisco San Francisco, CA United States; 2 Center for Vulnerable Populations at Zuckerberg San Francisco General Hospital University of California San Francisco San Francisco, CA United States; 3 School of Public Health Boston University Boston, MA United States; 4 University of California Berkeley, CA United States; 5 Department of Public Health California State University East Bay Hayward, CA United States; 6 Division of General Internal Medicine at Zuckerberg San Francisco General Hospital University of California San Francisco San Francisco, CA United States

**Keywords:** misinformation, COVID-19, South Asians, disparities, social media, infodemiology, WhatsApp, messages, apps, health information, reliability, communication, Asian, English, community, health, organization, public health, pandemic

## Abstract

**Background:**

South Asians, inclusive of individuals originating in India, Pakistan, Maldives, Bangladesh, Sri Lanka, Bhutan, and Nepal, comprise the largest diaspora in the world, with large South Asian communities residing in the Caribbean, Africa, Europe, and elsewhere. There is evidence that South Asian communities have disproportionately experienced COVID-19 infections and mortality. WhatsApp, a free messaging app, is widely used in transnational communication within the South Asian diaspora. Limited studies exist on COVID-19–related misinformation specific to the South Asian community on WhatsApp. Understanding communication on WhatsApp may improve public health messaging to address COVID-19 disparities among South Asian communities worldwide.

**Objective:**

We developed the COVID-19–Associated misinfoRmation On Messaging apps (CAROM) study to identify messages containing misinformation about COVID-19 shared via WhatsApp.

**Methods:**

We collected messages forwarded globally through WhatsApp from self-identified South Asian community members between March 23 and June 3, 2021. We excluded messages that were in languages other than English, did not contain misinformation, or were not relevant to COVID-19. We deidentified each message and coded them for one or more content categories, media types (eg, video, image, text, web link, or a combination of these elements), and tone (eg, fearful, well intentioned, or pleading). We then performed a qualitative content analysis to arrive at key themes of COVID-19 misinformation.

**Results:**

We received 108 messages; 55 messages met the inclusion criteria for the final analytic sample; 32 (58%) contained text, 15 (27%) contained images, and 13 (24%) contained video. Content analysis revealed the following themes: “community transmission” relating to misinformation on how COVID-19 spreads in the community; “prevention” and “treatment,” including Ayurvedic and traditional remedies for how to prevent or treat COVID-19 infection; and messaging attempting to sell “products or services” to prevent or cure COVID-19. Messages varied in audience from the general public to South Asians specifically; the latter included messages alluding to South Asian pride and solidarity. Scientific jargon and references to major organizations and leaders in health care were included to provide credibility. Messages with a pleading tone encouraged users to forward them to friends or family.

**Conclusions:**

Misinformation in the South Asian community on WhatsApp spreads erroneous ideas regarding disease transmission, prevention, and treatment. Content evoking solidarity, “trustworthy” sources, and encouragement to forward messages may increase the spread of misinformation. Public health outlets and social media companies must actively combat misinformation to address health disparities among the South Asian diaspora during the COVID-19 pandemic and in future public health emergencies.

## Introduction

Misinformation, or false and inaccurate information, is a major public health challenge during the COVID-19 pandemic. The World Health Organization has identified COVID-19 information as an “infodemic”—an overabundance of COVID-19–related information, including deliberate attempts to foment misinformation [1]. Many formal definitions of misinformation exist [2-4]; misinformation is sometimes distinguished from disinformation (ie, false information with intent to harm) and malinformation (ie, facts used out of context with intent to harm). For the purpose of this paper, we use the term “misinformation” as an umbrella term to comprise false information, where the intent is not apparent [5]. Social media is an important channel of distribution of COVID-19 misinformation; false news diffuses more quickly than truth [6], and low-credibility web sources are shared more frequently than any single high-credibility news source on Facebook or Twitter [7].

The South Asian diaspora, defined as communities with origins from Bangladesh, Bhutan, India, Maldives, Nepal, Pakistan, and Sri Lanka [8], is highly active on social media platforms. One common platform is WhatsApp. Unlike public social media platforms, such as Facebook, Twitter, and Instagram, WhatsApp is a private messaging platform allowing users to send information through text, photos, and videos to one person directly or in groups of up to 1023 individuals. Almost 400 million Asian Indians use WhatsApp, comprising the greatest number of platform users worldwide [9]. WhatsApp messages are readily forwarded with limited capacity to determine the original author or provide factual checks, enabling misinformation to spread easily.

Misinformation is harmful for public health. For example, myths circulating about various remedies, such as highly concentrated alcohol, ingested sanitizer, or Datura seeds led to cases of illness, blindness, and death [10]. Belief in COVID-19 vaccine conspiracy theories is associated with vaccine hesitancy [11]. Areas with higher exposure to news media denying COVID-19 severity are associated with greater COVID-19 case rates and deaths [12]. Given that misinformation related to COVID-19 may drive COVID-19 morbidity and mortality, understanding the ways COVID-19 misinformation spreads within specific communities is paramount. This is concerning given the disproportionate burden of COVID-19 infection, hospitalization [13], and death experienced by South Asians globally [14].

Understanding culturally specific misinformation may inform policy-making and targeted public health messaging efforts. As part of the COVID-19–Associated misinfoRmation On Messaging apps (CAROM.) study, we analyzed COVID-19 misinformation circulated within the South Asian diaspora via WhatsApp.

## Methods

### Procedure

**Figure 1:**
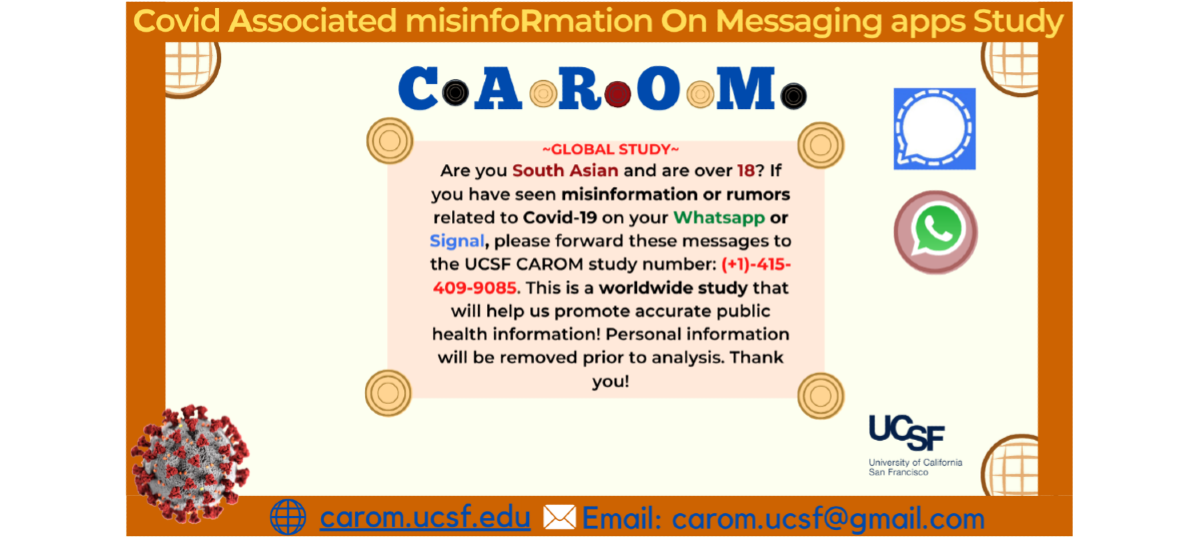
COVID-19–Associated misinfoRmation On Messaging apps (CAROM) Study web-based recruitment flyer.

Individuals who self-identified as members of the South Asian community older than 18 years of age anywhere globally were eligible for inclusion in the study. We specifically chose WhatsApp for sampling due to its significance as one of the most widely used messaging programs among the South Asian Diaspora [9]; it is therefore widely recognized and familiar in the South Asian community. We recruited participants via English-language outreach on Twitter, Facebook, and WhatsApp using web-based flyers with a QR code, hashtags, direct messages to community leaders, blog posts [15], and emails to South Asian organizations. We named the study after a beloved game that is familiar across the diaspora (Figure 1); additional recruitment materials are available in Multimedia Appendix 1.

We asked individuals who self-identified as members of the South Asian or Desi community to forward deidentified screenshots of WhatsApp messages containing what they perceived to be “misinformation or rumors” related to COVID-19 to an official study phone number. This allowed us to receive messages being transmitted or forwarded within WhatsApp without any personal or identifiable information included. We chose this method to allow individual WhatsApp users to share what was being transmitted in their private feeds in a deidentified manner, as a means to access traditionally closed communications in a way that protected the privacy of individual users. We requested WhatsApp messages with potential COVID-19 misinformation to be forwarded to an official study phone number. The research team advertised the study starting March 23, 2021, and collected messages until June 3, 2021. The team deidentified screenshots and media files if they were not already deidentified by the study participant. Study team members reviewed each message content to determine if inclusion criteria were met. Specifically, they assessed the relevance to COVID-19 and whether or not the information in the message was factual based on the team’s medical and scientific knowledge as well as web-based fact-checking when appropriate. Messages not in English; received after June 3, 2021; not relevant to COVID-19; or not containing misinformation were excluded.

The team developed an abstraction form to identify media format (eg, written text, picture, video, URL, or a combination of these formats), country or location mentioned, content category, and tone in REDCap (Research Electronic Data Capture; Vanderbilt University; Multimedia Appendix 2). Two team members (KK and KP) abstracted the messages and conducted open coding, using content analysis of messages and categorizing messages into thematic groups of specific “content types” of COVID-19 misinformation. The entire research team cross-checked coding and the thematic analysis until the team arrived at consensus.

### Ethics Approval

This study was approved by the Internal Review Board of University of California, San Francisco (20-32758).

## Results

We collected 108 messages, deduplicated to 96, of which 55 messages met the inclusion criteria. Message formats included plain text, images, and videos, or a combination of these formats (Table 1). India was most commonly mentioned (21/55, 38%); followed by China (7/55, 13%), the United States (4/55, 7%), and Italy (3/55, 5%).

The content fit into one or more of the following thematic categories: (1) community transmission, (2) prevention, (3) treatment, and (4) products (Table 2). “Community transmission” messages included conspiracy theories about the origins and spread of COVID-19; one example described India’s second wave as suspicious following rising political tensions with China. Many “prevention” messages proposed ways to prevent coronavirus infection through home-based strategies, such as inhaling steam, eating a banana every day, or consuming alkaline foods. Messages about “treatment” offered self-treatments for COVID-19, such as drinking “a teaspoon of pepper powder, two teaspoons of honey, and ginger juice.” Lastly, messages about “products” publicized commercial treatments or cures for COVID-19, such as a nasal spray purported to offer protection from SARS-CoV-2.

Messages ranged from containing “universal” misinformation addressed to the general public to “South Asian–specific” content containing cultural references. One universal message was a video of an alleged Irish scientist describing purported mortality risk with messenger RNA vaccines. In comparison, a South Asian–specific reference discussed traditional natural remedies, such as Ayurveda and homeopathy. A few messages appealed to ethnic or national pride, with statements such as “we Indians are built to last” or “proud to be an Indian.”

Some messages contained information that was entirely false; for example, one image claiming to be published by UNICEF (United Nations International Children’s Emergency Fund) stated that the coronavirus will be killed if it is exposed to a temperature of 26-27 °C, and therefore, encouraged drinking hot water and increasing sun exposure. However, other messages shared a mixture of true and false information. One message combined evidence-based recommendations for preventing COVID-19 infection, such as social distancing and wearing a face mask, while also encouraging behaviors without evidence, such as eating vegetarian food and removing belts and rings.

Message tone included fear- or panic-based encouragement to share purportedly useful information and pseudoscientific expertise. Pseudoscientific messages contained scientific jargon unfamiliar to a nonscientific audience, such as “anticoagulants” and “ground-glass opacities,” in combination with references to reputable organizations, such as the World Health Organization and the Indian Council on Medical Research, as well as individual experts, such as doctors and scientists. A third of messages (18%-33%) used a pleading or encouraging tone to promote dissemination, asking recipients to “share with all your family and friends” or “send to all your groups.”

**Table 1:** Summary characteristics of the sample (N=55).

Characteristics		Values, n (%)
**Message format**		
	Text only	32 (58)
	Image only	15 (27)
	Video only	13 (24)
	Link	6 (11)
	Other	1 (2)
**Countries mentioned**		
	India	21 (38)
	China	7 (13)
	United States	4 (7)
	Italy	3 (5)
	Japan	2 (4)
	Australia	1 (2)
	Bangladesh	1 (2)
	Bhutan	1 (2)
	Ireland	1 (2)
	Nepal	1 (2)
	Pakistan	1 (2)
	Spain	1 (2)
	Sri Lanka	1 (2)
	Switzerland	1 (2)
	Taiwan	1 (2)
**Tone**		
	Good intentions	27 (50)
	Pleading or call to action	18 (33)
	Warning or fear-based	10 (18)
	Blame	4 (7)
	Other	6 (15)

**Table 2:** Main thematic domains of misinformation.

Level	Domains	Subthemes	Definition	Examples
**Individual**				
	Prevention or diagnosis	Self-diagnosis and self-remedies	Content had to do with preventing COVID-19 disease, exposure to Sars-CoV-2, and screening for COVID-19.	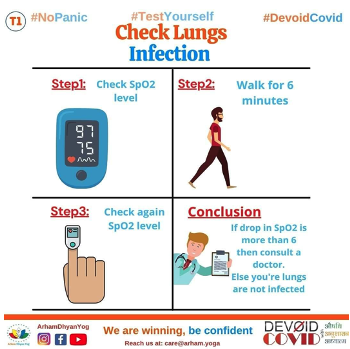 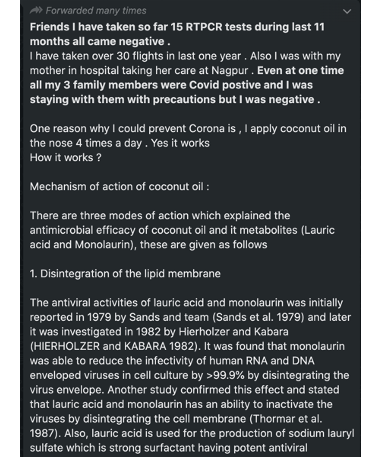
	Treatment	Ayurveda, homeopathy, or natural remedies	Content promoting nonevidence-based means to treat or cure COVID-19	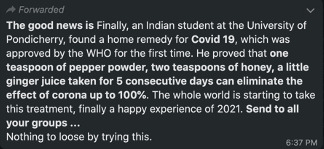
	Products or services		For-sale devices or products marketed to reduce risk, prevent, or treat COVID-19 infection	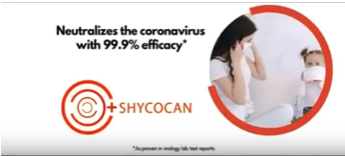 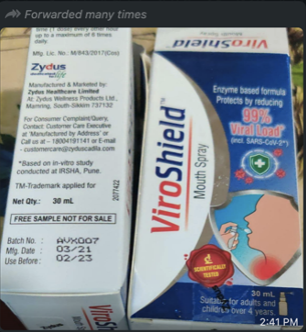
**Population**				
	Community transmission	Conspiracy theories	Content explains how and why COVID-19 is spreading at the local or international level	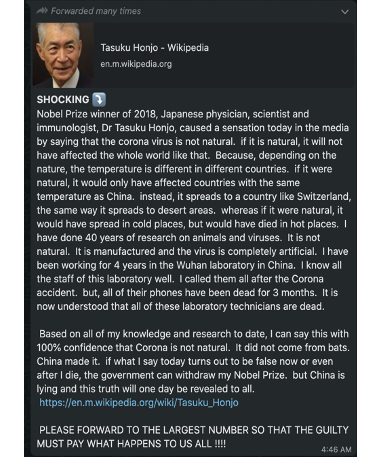
	Cultural pride		Speaking to positive or robust aspects of South Asian identity, value of South Asian attributes, or direct contributions to efforts to fight COVID-19	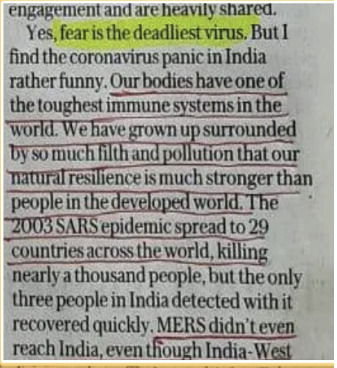

## Discussion

### Principal Findings

We found misinformation circulating through South Asian networks is largely aimed at providing alternative explanations for the etiology and spread as well as alternative treatment and prevention methods for COVID-19.

The South Asian diaspora comprises the largest diaspora population in the world [16], with high social media use, and transnational communication via messaging means that misinformation can have global reach almost instantaneously. Our analysis adds to the literature by providing a window into the nature of these closed group conversations. A prior narrative review of misinformation across Asian American communities [17] and a study of Twitter misinformation in Hindi [18] both highlighted religious-based content, including Islamophobic messaging, as the major thematic finding for South Asians. We instead found a focus on understanding, preventing, and treating the spread of the virus using alternative or Ayurvedic methods. Our study is the first to our knowledge to assess this topic within the broader global South Asian diaspora.

COVID-19 misinformation, disinformation, and malinformation have persisted due to a perfect storm of evolving uncertainty of the disease, malintent of actors pushing political or business interests, and the well-meaning intentions of community members who may have low health or media literacy [19]. The South Asian community has experienced a disproportionate burden of COVID-19 with less media attention, particularly extensive collective trauma after the 2021 Delta variant surge, which may have killed more than 3 million people [20]. South Asian audiences may thus be particularly receptive to promoting messages that provide a sense of clarity, trustworthiness, and personal control in an uncertain time.

Misinformation circulates more readily among homogenous groups or “echo chambers” [21], and misinformation with culturally specific language to promote “in-group identity” may receive higher engagement [22,23]. Messages broadcasting cultural pride may therefore be more readily amplified within relatively insular groups of South Asian users. Messages included “name checks” and logos of reputable organizations or individuals, enhancing trust [24]. Factual information was blended with false statements, mimicking credibility. Lastly, participants were often exhorted to share messages with others, encouraging the spread of misinformation under the well-meaning intention of promoting community safety.

The closed, trusted groups in WhatsApp and similar platforms, such as Viper, Weibo, and Signal, may actually foment misinformation [25,26]. WhatsApp currently puts the burden on individual users to stop its spread [27]. In epidemiological modeling, limits on the number of message forwards slows the spread of misinformation but does not ultimately stop the spread of viral content [28]. Clear countermessaging to identify and correct misinformation can be effective [29]. when promoted across a multitude of platforms; however, more research is needed to identify how to best countermessage COVID-19–related misinformation without causing unintended backfire [30,31]. Social media corporations must do more to monitor, detect, and possibly delete or flag dangerous misinformation. Fact-checking organizations can also perform this role and circulate countermessaging [32,33]. South Asian–specific, culturally literate public health experts and community organizations should collaborate with Fact-checking organizations to create and disseminate countermessaging.

Given the complexity of misinformation, multiple counteracting strategies beyond countermessaging will be needed. This will include the use of machine learning to flag or identify misinformation more easily, policy changes to increase legal accountability for harmful misinformation, and heightened scrutiny and investigation of organizations and web-based influencers who are frequent sources or spreaders of misinformation [30].

These web-based myths can have very real consequences. The spread of culturally specific misinformation may lead to unsafe health behaviors [34] and contribute to preventable burdens of COVID-19 among South Asian communities [35,36]. Given the vast diversity of ethnic groups included by the term “Asian,” more funding and research to promote disaggregated data collection and analysis (eg, for specific East Asian, South Asian, Southeast Asian, and other groups) [37] is greatly needed to understand and hopefully counteract culturally specific misinformation regarding COVID-19 and future pandemics.

### Strengths and Limitations

Study strengths include a transnational sample and focus on a social media platform not publicly visible for analysis. We were, however, unable to collect demographic information about original message senders or the recipients who forwarded the messages. We relied on participants to self-identify as members of the South Asian diaspora; participants may have had varying definitions of this identity. Our focus on English-language messages likely limited the content of the final sample, given the linguistic diversity of the South Asian diaspora. Our study focused on adult participants who could give informed consent; therefore, our analytic sample may not be generalizable for COVID-19 misinformation disseminated among children and adolescents. We could not gauge whether specific messages were shared with intent to harm, as with disinformation or malinformation, for purposes of “collective fact-checking” [38], or shared because they were genuinely believed. As our data collection relied on users identifying potential misinformation, our team may have not received the misinformation that users viewed in their WhatsApp feed but believed to be true. A potential limitation of our methods is that although the individual participant consented to share anonymous content from their WhatsApp, there were no means to obtain permission for analysis from the original poster or others who had forwarded the message. We ensured both the individual participant and any other WhatsApp users had zero identifiable information collected. Moreover, WhatsApp does not have any way to identify original posters, nor prior forwarders, of a specific message. Although our ethical approach was to maximize public health benefit and avoid any individual harm, interdisciplinary gold standards for social media research are still needed [39]. Given that messages were sent on a voluntary basis from individuals who chose to participate, this research cannot be viewed as a systematic analysis of misinformation shared across the South Asian diaspora on private messaging platforms. The sample should be considered hypothesis generating; larger samples may provide greater insights into messaging among the global South Asian diaspora.

### Conclusions

We found that COVID-19–related misinformation from WhatsApp messages within the South Asian diaspora relate to four themes: transmission, prevention, treatment, and product or service promotion. Tactics to enhance credibility and spread of messages included use of jargon, blending of true and false information, mention of reputable organizations and expert credentials, and references to ethnic pride. Encouragement to share misinformation messages among personal networks makes it urgent to find ways to interrupt misinformation in real time so as not to exacerbate COVID-19 disparities. Novel public health strategies, including culturally specific fact-checking, will be needed to counteract misinformation among the South Asian diaspora.

## Appendix

Multimedia Appendix 1 Recruitment materials for COVID-19–Associated misinfoRmation On Messaging apps (CAROM) Study.

Multimedia Appendix 2 Abstraction form.

## Abbreviations

CAROM: COVID-19–Associated misinfoRmation On Messaging apps
UNICEF: United Nations International Children’s Emergency Fund
