# Supplementary material for: COVID-19–Associated Misinformation Across the South Asian Diaspora: Qualitative Study of WhatsApp Messages
Source: JMIR Infodemiology. 2023 Jan 5;3:e38607. doi: 10.2196/38607 (PMC10013129; doi:10.2196/38607)
Supplement: Multimedia Appendix 1 [file infodemiology_v3i1e38607_app1.docx]

Appendix 1 Recruitment Materials

Email

Dear Professors, Colleagues, and Friends,

I hope you all are doing well. This is Kiran Khosla, Boston College, 2019. I would like to inform you about a project I am working on that is relevant to you based on your background or research interests.

A team of researchers from the University of California, San Francisco **is collecting Whatsapp messages to examine the spread of misinformation related to the COVID-19 pandemic among the South Asian community worldwide**.

#### The Covid Associated misinfoRmation On Messaging apps (C.A.R.O.M.) study is a qualitative study documenting and assessing misinformation about Covid-19 over Whatsapp in South Asian communities.

#### Free messaging apps like WhatsApp have connected people across the globe; nearly 20 percent of the 2 billion Whatsapp users are located in India alone. However, it can be difficult to regulate misinformation on these apps given their private nature. Addressing misinformation about COVID-19 —the disease, treatment, vaccines, and government response—  is essential to supporting public health interventions during the pandemic. Given the absence of disaggregated data about the impact of misinformation on the South Asian community in particular, this study aims to shed light on the misinformation and rumors spreading through the South Asian diaspora and how the community responds to it.

Eligibility and participation:

#### South Asian (can live anywhere globally)

#### Over the age of 18

#### Seen rumors or misinformation about COVID-19, the vaccine, or anything related to the pandemic over WhatsApp

#### If you have seen misinformation in your communities over Whatsapp, please screenshot or forward these messages to 415-409-9085. Even if you aren’t sure, send it. We won’t be saving any of your personal information.

The study flyer and a WhatsApp message template is attached. For more information, please visit our [website](http://carom.ucsf.edu), [Facebook page](http://facebook.com/caromstudy), or email us at [carom.ucsf@gmail.com](mailto:carom.ucsf@gmail.com). Please share this information

with friends, family, and colleagues. Thank you!

Best,

Kiran Khosla

To South Asian organizations:

Hi ____,

I hope you are well! My name is Kiran Khosla, and I provided consultation on the SAALT Covid-19 survey.

I wanted to spread the word about the CAROM (Covid Associated misinfoRmation On social Messaging apps) study out of UCSF.

This study specifically explores COVID-19 related misinformation disseminated across the South Asian Diaspora via WhatsApp, to look for possible links between social media related misinformation and South Asian health disparities.

If you or someone you know is part of the South Asian/Desi Community, please spread the word across your networks!

We are collecting de-identified forwards and screenshots to the official CAROM account at 415-409-9085.  Feel free to email me directly with Q's.

Here is the link to our [tweet](https://twitter.com/UrmimalaSarkar/status/1374457647496273927?s=20)! I attach the study flyer to this email.

Thank you!

Best,

Kiran

**Whatsapp Message**


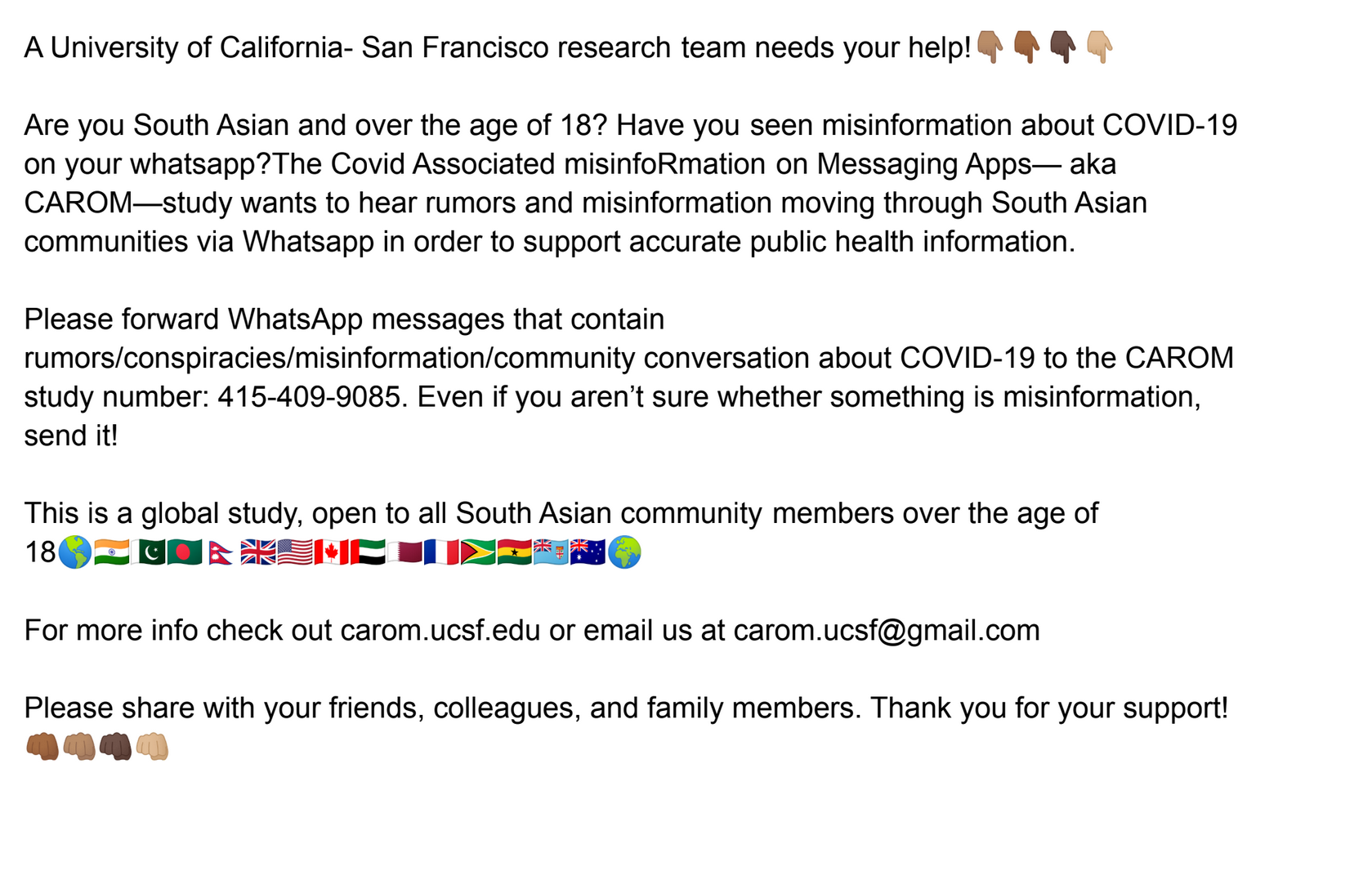


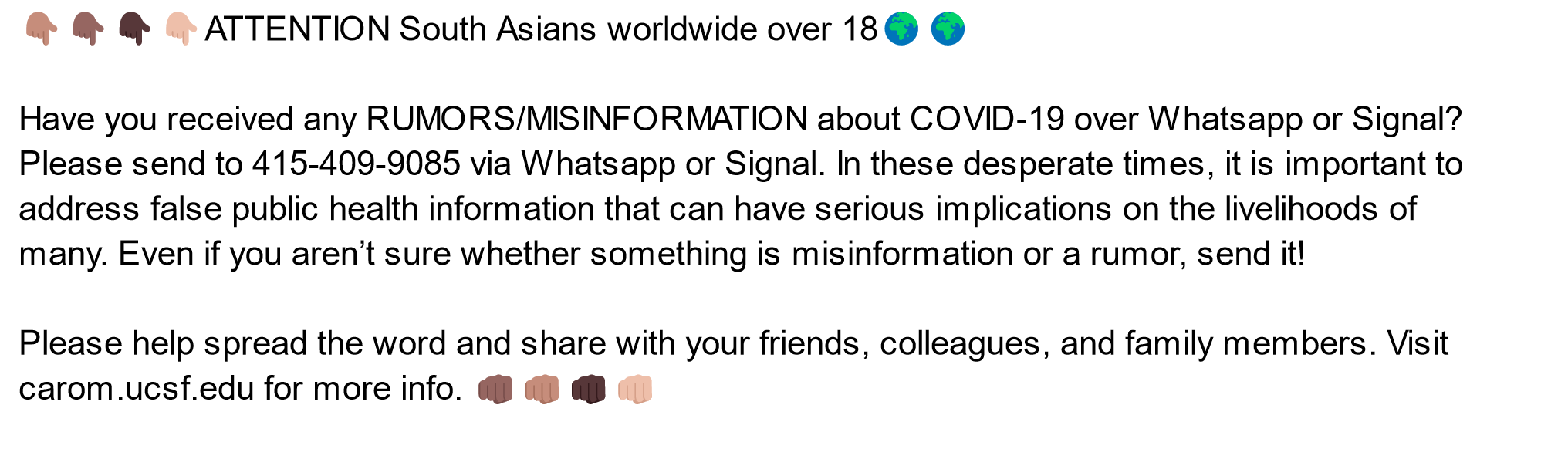


**Twitter**


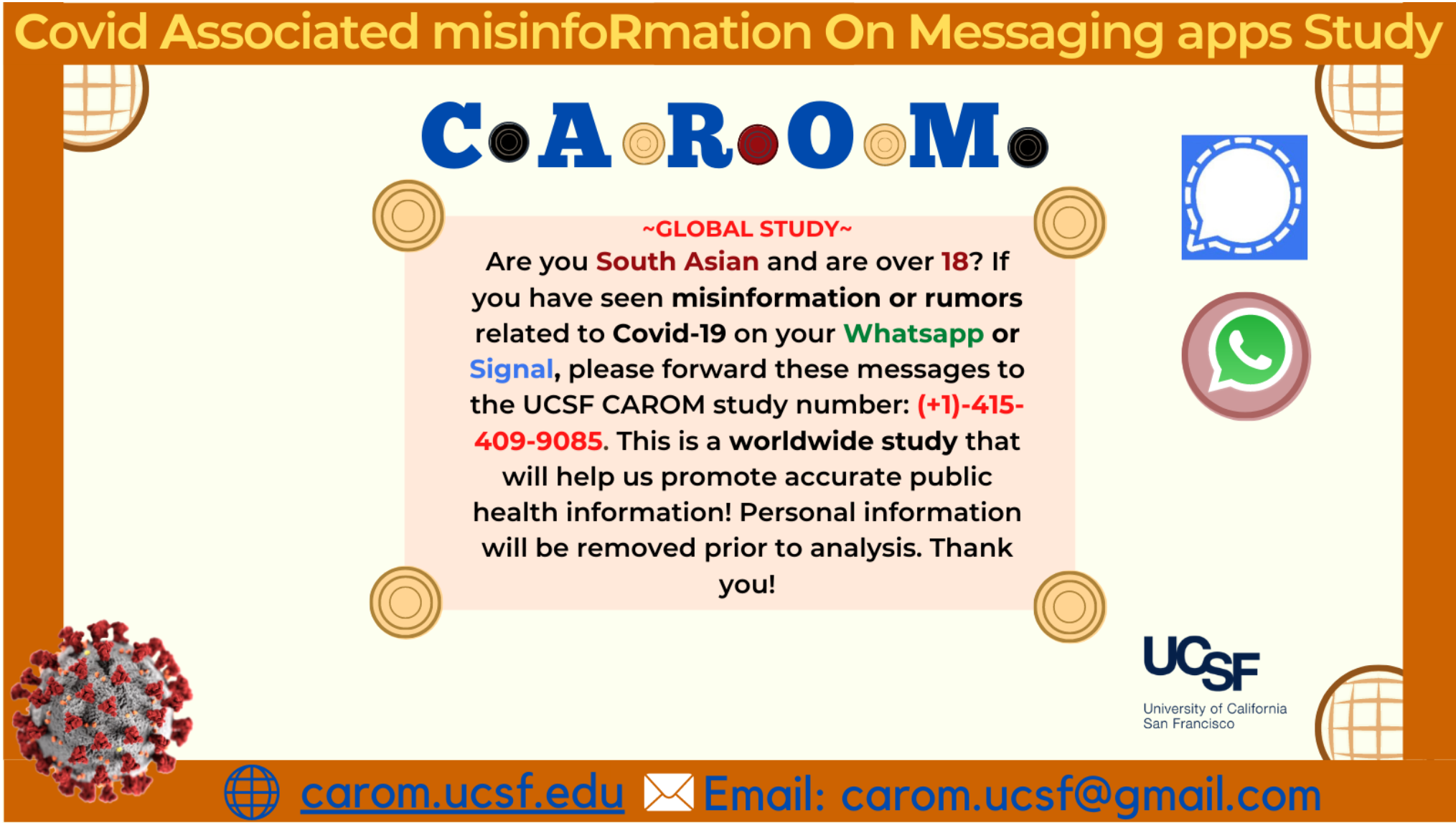


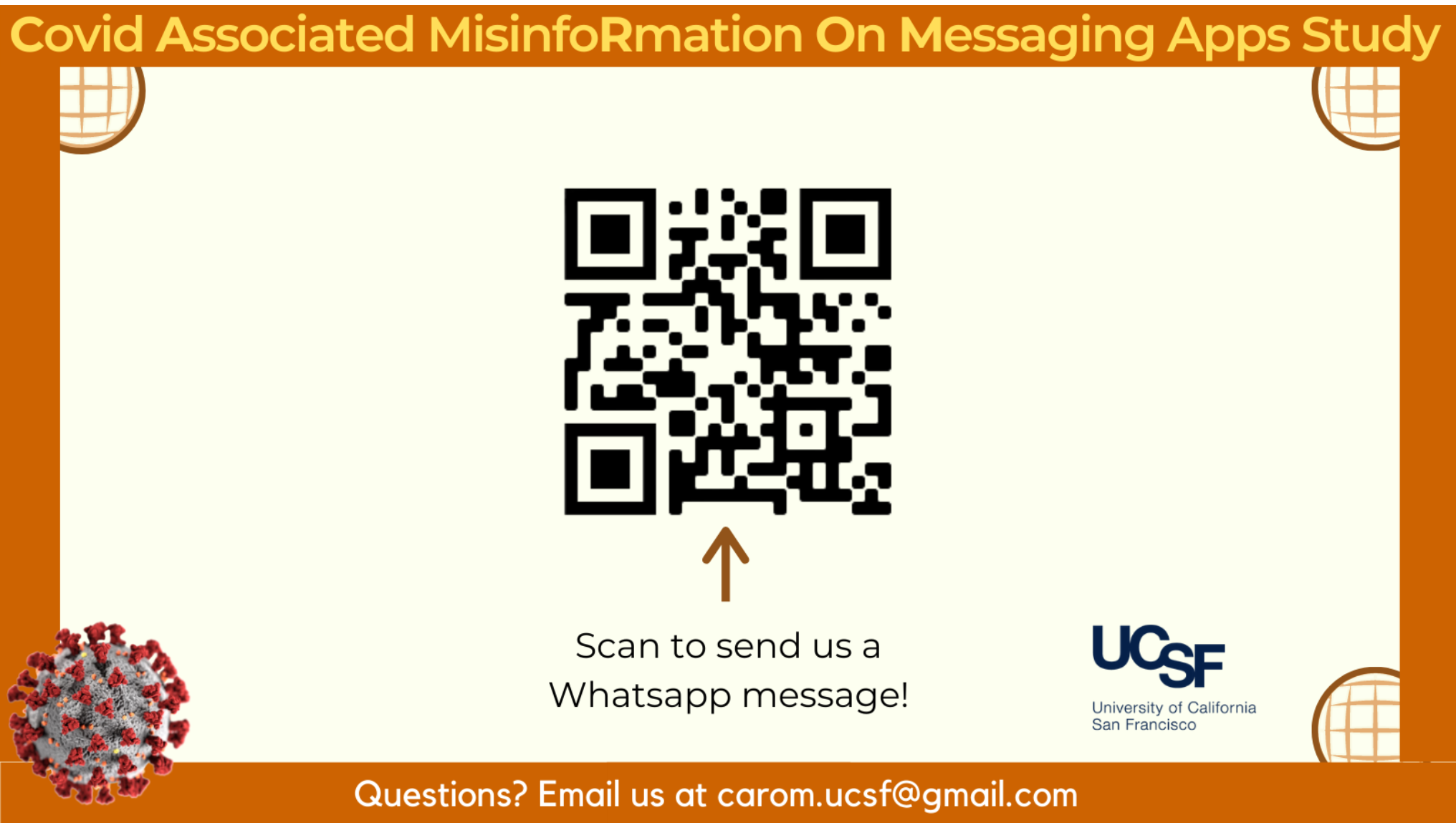


**Blog post:**

<https://redwhiteandbrown.substack.com/p/viral-virus-texts>
