# Supplementary material for: COVID-19–Associated Misinformation Across the South Asian Diaspora: Qualitative Study of WhatsApp Messages
Source: JMIR Infodemiology. 2023 Jan 5;3:e38607. doi: 10.2196/38607 (PMC10013129; doi:10.2196/38607)
Supplement: Multimedia Appendix 2 [file infodemiology_v3i1e38607_app2.pdf]

Form 1

Record ID	______________________
Participant (Sender) ID	______________________
Name(s) of file(s) in GDrive	______________________<br>(This is to keep track and make it easier to find)
Date received	______________________<br>(when the message was received)
Inclusion in Study	☐ Yes<br>☐ No<br>☐ Not sure- want to discuss<br>☐ Memo<br>(will this message be included?)
Inclusion in Study memo	______________________<br>(memo for inclusion variable)
Exclusion Reason	☐ Not English<br>☐ Not misinformation<br>☐ Not South Asian<br>☐ Not adult sender (18+)<br>☐ Does not seem Covid-19 related<br>☐ Other<br>☐ Memo<br>(Flexibility in whether msg is 'Covid-19 related')
Exclusion reason (if 'Other')	______________________
Exclusion memo	______________________
Messaging platform	◯ Whatsapp<br>◯ Signal
Type of Media- Check all that apply	☐ text<br>☐ image<br>☐ video<br>☐ link<br>☐ powerpoint<br>☐ voice memo<br>☐ other<br>☐ memo
Type of Media (if 'Other')	______________________

Type of Media memo	__________________________________
Link URL	__________________________________<br>(If the message contains a link)
Mentioned a specific location? Check all that apply.	☐ Country<br>☐ City<br>☐ Town/Village<br>☐ N/A<br>☐ Other<br>☐ Memo<br>(Are people talking about COVID broadly or locally?)
Mentioned a specific location (if 'Other')	____
Mentioned a Specific Location Memo	____<br>(memo for location variable)
Country- Check all that apply	☐ India<br>☐ Pakistan<br>☐ Nepal<br>☐ Bangladesh<br>☐ Sri Lanka<br>☐ Maldives<br>☐ United State of America<br>☐ Canada<br>☐ United Kingdom<br>☐ France<br>☐ Spain<br>☐ China<br>☐ Italy<br>☐ Afghanistan<br>☐ Bhutan<br>☐ Other<br>☐ Memo<br>(Are any of these countries mentioned? In text/images/emojis?)
Country (if Other)	__________________________________
Countries Mentioned Memo	__________________________________
City/cities mentioned	__________________________________<br>("mentioned" is broad)
Town(s)/village(s) mentioned	__________________________________

Individual(s), organization(s), institution(s) mentioned/referenced in message, not including author of the post<br><br>Check all that apply	☐ Individual<br>☐ Organization<br>☐ Government<br>☐ University<br>☐ Religious institution<br>☐ Company/business<br>☐ Other<br>☐ N/A<br>☐ Memo<br>("character(s)"/"stakeholder(s)" mentioned in message not including author of the post. Use the memo section for multiple stakeholders)
Individual/Organization/Institution Referenced in Message (if 'Other')	____________________________<br>(ex) soccer team, cricket team; include occupation/title if applicable)
Individual(s)/organization(s)/institution(s) memo	__________________________________
Type of Individual(s)<br><br>private=anyone who described as a family member, neighbor, friend, or partner<br><br>public=anyone who is not described as a family member or neighbor or friend or partner; someone referenced by their occupation/role in society outside of personal relationships<br><br>Check all that apply	☐ Self<br>☐ Private<br>☐ Public figure<br>☐ Other<br>☐ Unable to discern<br>☐ Memo<br>(If an individual is mentioned, are they a private individual-- described by personal relationships? Are they a public-facing professional? public=anyone who is not described as a family member or neighbor or friend or partner; someone referenced by their occupation/role in society outside of personal relationships)
Type of Individual (if 'Other')	__________________________________
Type of Individuals Memo	__________________________________
If 'self', describe how the person talks about themselves	__________________________________<br>(personal experience, patient experience, setting, occupation)

Type of Individual: Public figure(s)<br><br>Check all that apply.	☐ Someone with a "Dr." in their title<br>☐ Academic<br>☐ Doctor/healthcare provider/medical professional<br>☐ Public Health Professional<br>☐ Government official<br>☐ religious/cultural leader<br>☐ influencer<br>☐ Celebrity<br>☐ Media/journalist<br>☐ Other<br>☐ Memo<br>(public=anyone who is not solely described as a family member or neighbor or friend or partner; someone referenced by their occupation/role in society outside of personal relationships. Can select 'self' and 'public figure' options)
Public Figure (if 'Other')<br>-type of individual	______________________________
Public Figure Memo<br>-type of individual	______________________________
Author(s) of the post<br><br>Check all that apply.	☐ Academic<br>☐ Doctor/healthcare provider/medical professional<br>☐ Someone with the "Dr." title<br>☐ Public Health Professional<br>☐ Government official<br>☐ Religious/Cultural leader<br>☐ Influencer<br>☐ Celebrity<br>☐ Media/journalist<br>☐ Specific person (name given)<br>☐ Organization<br>☐ Government<br>☐ University<br>☐ Religious institution<br>☐ Company/business<br>☐ Other<br>☐ No author<br>☐ Unable to discern author<br>☐ memo<br>(Does the post have author(s)? Someone taking credit? Supposed authors. This does not mean original sender)
Author(s) of the Post (if 'Other')	______________________________
Author(s) of the Post memo	______________________________
File upload	(attach message(s))
Link to gdrive file	________________________

Message Content/Description

____

Content Category- check all that apply

☐ Treatment
☐ Personal Prevention
☐ Products
☐ Cause of disease in person
☐ Meme/joke/entertainment
☐ Possibly not misinformation
☐ Spread at the population level
☐ Conspiracy theory / suspected conspiracy theory
☐ Other
☐ Memo
(Treatment option has branching logic, personal prevention has branching logic. Add comment if warrants further discussion)

Content Category (if 'Other')

____

Content Category memo

____

Treatment

☐ Cures
☐ Vaccine
☐ Home remedy
☐ Ayurveda
☐ Product
☐ Other
☐ Memo

Treatment (if 'Other')

____

Treatment memo

____

Personal Prevention

☐ Memo
☐ Ayurveda
☐ Steam
☐ Products
☐ Prayers
☐ Other
(prevent contracting the disease)

Personal prevention memo

____

Personal Prevention (if 'Other')

____

Forwarded Many Times	☐ Forwarded<br>☐ Forwarded many times<br>☐ Screenshot/ other media type sent and does not include<br>☐ No indication<br>☐ Memo<br>(Does this message have the forwarded many times indicator? Only for Whatsapp messages)
Memo for "Forwarded many times"	__________________________________
Misinformation assessment	☐ Partially accurate but misleading<br>☐ Partially accurate<br>☐ Accurate but misleading<br>☐ Misleading<br>☐ N/A<br>☐ Other<br>☐ Not sure<br>☐ Not sure, want to discuss<br>☐ Memo<br>(Capturing the gray area of misinformation. Don't do much outside research?)
Misinformation measure (if 'Other')	__________________________________<br>(Capturing the gray area of misinformation, describe as best you can)
Misinformation Assessment Memo	____
Tone- check all that apply.	☐ Warning/fear-based<br>☐ Conspiracy theory<br>☐ Good intentions, trying to help<br>☐ Ayurveda/traditional healing practices<br>☐ Pleading/call to action<br>☐ Blame<br>☐ Unscientific beliefs<br>☐ None<br>☐ Other<br>☐ unable to discern<br>☐ memo<br>(what kind of feelings does the message elicit?)
Tone memo	____
Tone (if 'other')	__________________________________
Use of scientific jargon, images, labels, symbols?	◯ Yes<br>◯ No<br>(written, labels)

List scientific jargon, images, labels, symbols	__________________________________
Unverified endorsements	__________________________________<br>(includes endorsements written in text, images)
Additional Notes	__________________________________
Who coded	◯ KP<br>◯ KK
Quoteworthy?	◯ Yes<br>◯ No<br>(would this message be worth using an example of a particular content category?)
